# Cleveland Clinic Cognitive Battery (C3B): Normative, Reliability, and Validation Studies of a Self-Administered Computerized Tool for Screening Cognitive Dysfunction in Primary Care

**DOI:** 10.3233/JAD-220929

**Published:** 2023-04-04

**Authors:** Stephen M. Rao, Rachel Galioto, Megan Sokolowski, Madelyn Pierce, Lisa Penn, Anna Sturtevant, Blazenka Skugor, Brent Anstead, James B. Leverenz, David Schindler, David Blum, Jay L. Alberts, Lori Posk

**Affiliations:** a Lou Ruvo Center for Brain Health, Neurological Institute, Cleveland Clinic, Cleveland, OH, USA; b Mellen Center for Multiple Sclerosis Treatment and Research, Neurological Institute, Cleveland Clinic Foundation, Cleveland, OH, USA; c Twinsburg Family Health and Surgery Center, Internal Medicine and Geriatrics Department, Twinsburg, OH, USA; d Family Medicine, Hillcrest Hospital, Cleveland Clinic, Mayfield Heights, OH, USA; e Department of Biomedical Engineering, Lerner Research Institute, Cleveland Clinic, Cleveland, OH, USA; f Qr8 Health, Inc., Cleveland, OH, USA; g Health and Wellness Center, Cleveland Clinic, Vero Beach, FL, USA

**Keywords:** Cognitive Assessment Screening Instrument, mild cognitive impairment, Mini-Cog, neuropsychological testing, primary health care, regression analysis, test-retest reliability

## Abstract

**Background:**

The self-administered iPad-based *Cleveland Clinic Cognitive Battery (C3B) was* designed specifically for the efficient screening of cognitive functioning of older adults in a primary care setting.

**Objective:**

1) Generate regression-based norms from healthy participants to enable demographic corrections to facilitate clinical interpretation; 2) estimate test-retest reliability and practice effects; 3) examine ability to discriminate mild cognitive impairment (MCI) from healthy aging; 4) d etermine validity of screening in a distracting clinical environment; and 5) determine completion rates and patient satisfaction in a primary care setting.

**Methods:**

Study 1 (*S1*) recruited a stratified sample of 428 healthy adults, ages 18–89, to generate regression-based equations. *S2* assessed 2-week test-retest reliability and practice effects in 30 healthy elders. *S3* recruited 30 MCI patients and 30 demographically-matched healthy controls. In *S4,* 30 healthy elders self-administered the C3B in a distracting environment and in a quiet private room in counterbalanced order. In a demonstration project, 470 consecutive primary care patients were administered the C3B as part of routine clinical care (*S5*).

**Results:**

C3B performance was primarily influenced by age, education, and race (*S1*), had acceptably high test-retest reliability and minimal practice effects (*S2*), discriminated MCI from healthy controls (*S3*), was not negatively impacted by a distracting clinical environment (*S4*), had high completion rates (>92%) and positive ratings from primary care patients (*S5*).

**Conclusion:**

The C3B is a computerized cognitive screening tool that is reliable, validated, self-administered, and is conducive to integration into a busy primary care clinical workflow for detecting MCI, early Alzheimer’s disease, and other related dementias.

## INTRODUCTION

Mild cognitive impairment (MCI), a significant risk factor for Alzheimer’s disease (AD), is estimated to be present in 15–20% of persons age 65 and older [1], equating to 11.6 million US citizens. Although MCI can occur for reasons other than AD and some MCI patients remain stable or revert to normal cognition, approximately 32–38% of MCI patients convert to dementia within a 5-year follow-up [2, 3]. A substantial proportion of MCI and early AD patients are underdiagnosed and underreported, with an AD diagnosis typically occurring late in the AD disease course [4]. A 2018 report by the Alzheimer’s Association [4] indicates that an early and accurate diagnosis of MCI and early stage AD has substantial medical, emotional, social, and economic benefits. Using a model in which 100% of AD patients receive a diagnosis during the MCI stage, a total savings of $7.9 trillion (2017 dollars) in health care spending would be realized. In addition, an early diagnosis of MCI maximizes the chances of participation in a clinical trial. Finally, a 2017 RAND Corporation report [5] indicated that the US health-care system is unprepared to cope if a disease-modifying drug is approved, with a major constraint being the limited capacity to identify MCI and early-stage AD patients. With the recent FDA approval of aducanumab (Aduhelm), this hypothetical scenario is an emergingreality.

A growing consensus has coalesced on the view that the identification of the earliest stages of AD should occur in the primary care clinic [6]. A 2020 report of the Lancet Commission has identified 12 modifiable risk factors that account for 40% of all dementias [7]. Many of these risk factors, such as hypertension, obesity, smoking, hearing impairment, depression, physical inactivity, and diabetes, are amenable for primary care intervention that could delay or prevent dementia if detected in the earliest stage of disease [6, 7].

Currently, there are no definitive screening tests for MCI and early AD for healthy elders. Imaging (PET) and cerebrospinal fluid/blood-based biomarkers have evolved for detecting AD pathophysiological processes [8]. However, because these pathological changes occur 10–20 years prior to cognitive symptoms, routine screening would result in high false positive rates. Lack of definitive results, coupled with the high costs of these approaches, make wide scale screening for MCI/early AD using current measures impractical.

In contrast, wide scale cognitive screening in a primary care setting offers a low-cost alternative. In dementia clinical practice, there are numerous validated cognitive screening tests: Mini-Mental State Examination (MMSE) [9], Modified Mine Mental State (3MS) [10], Montreal Cognitive Assessment (MoCA) [11], and the St. Louis University Mental Status [12]. Typically, such tests are administered by clinicians to patients already experiencing cognitive symptoms or have subjective cognitive complaints. Such tests are impractical for wide scale cognitive screening since the administration and scoring times typically exceed 10 min. In contrast, the Mini-Cog [13] requires only 3 min to administer so could serve as a useful wide scale screening tool. An alternative approach is to screen for cognitive impairment using self-administered, computerized tests. A 2017 NIA workshop [14], “Cost-Effective Early Detection of Cognitive Decline”, recommended integration of validated, self-administered computerized cognitive screening tools into the primary care clinical workflow for detecting MCI, early AD, and other related dementias.

To meet this need, we developed the Cleveland Clinic Cognitive Battery (C3B), a brief (10-min), low-cost, self-administered, digital cognitive assessment battery, specifically designed for efficient screening of cognitive impairment of older adults in a primary care setting. The C3B consists of two measures, the Visual Memory Test (VMT), a test of episodic memory, and the Processing Speed Test (PST), a measure of information processing and sustained attention [15]. A detailed description of the C3B test modules is provided in the Methods.

Here, we provide the results of five systematic studies detailing the continuum of C3B validation to initial implementation. The *first* describes a normative study designed to generate regression-based equations derived from healthy participants that adjust raw scores based on age, education, sex, and race. From these equations, z-scores can be calculated to facilitate clinical interpretation. The *second* assesses two-week test-retest reliability and practice effects; high reliability and low practice effects are important for interpreting the significance of longitudinal changes in cognition. The *third* study examines the ability of the C3B to discriminate MCI patients from demographically-matched healthy individuals; in addition, using signal detection methodology, we compare the strength of C3B discriminability with that of 1) standardized paper-and-pencil neuropsychological tests used to diagnose MCI/AD and 2) a test frequently used to screen cognitive function in a primary care setting (Mini-Cog). The *fourth* study was designed to determine whether valid C3B test results could be obtained in a typical primary care clinic waiting room with distracting sights and sounds. The *fifth* study involved the implementation of the C3B in a primary care setting to determine: 1) PST and VMT completion rates; 2) patient satisfaction with C3B cognitive screening, and 3) the relationship between subjective memory complaints and objective C3B cognitive test findings.

## CLEVELAND CLINIC COGNITIVE BATTERY

The 10-min C3B screening battery consists of two test modules: VMT (episodic learning and delayed memory) and the PST (information processing speed and incidental memory). Both modules resemble standardized, technician-administered neuropsychological tests used in clinical practice: Brief Visuospatial Memory Test-Revised [16] and Symbol Digit Modalities Test [17], respectively. It is important to note that these technician-administered clinical neuropsychological tests of episodic memory and processing speed are routinely administered as part of the clinical work-up of a patient suspected of having MCI or early AD.

Both iPad modules use standardized auditory and on-screen visual test instructions and stimuli consisting of symbols and single digits; thus, the screening battery can be adapted to multiple cultures with appropriate translations of the verbal instructions. Test administration materials include the iPad and a set of noise-cancelling, over-the-ear headphones. Below is a description of each of the C3B tests:

### Visual memory test

**Fig. 1:**
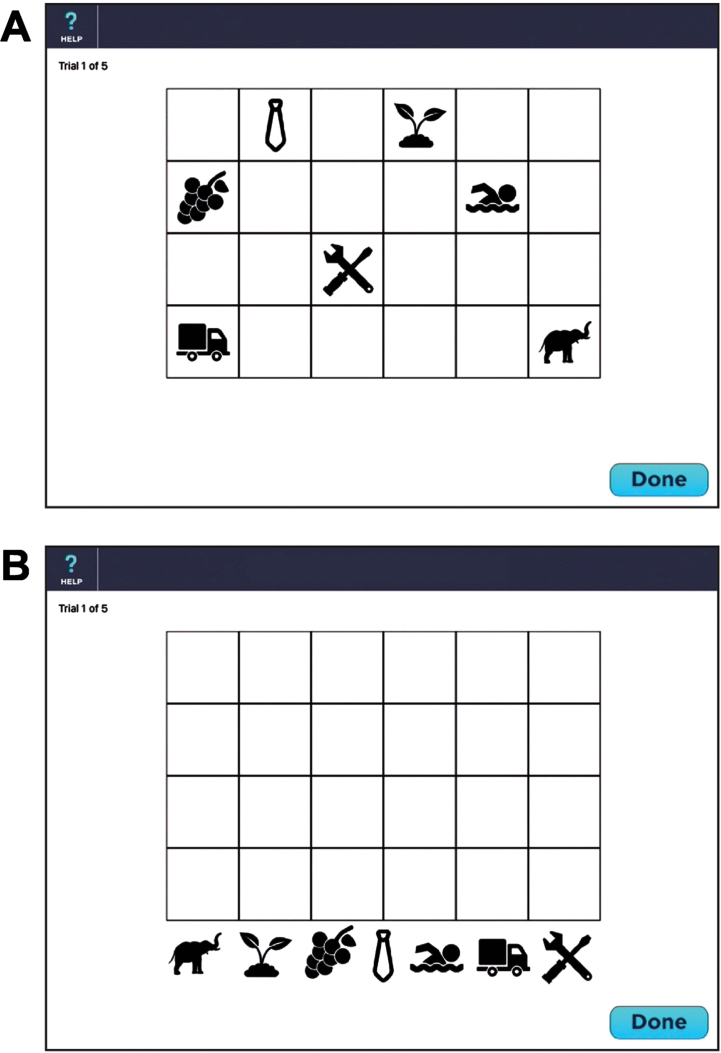
Screenshot of the Visual Memory Test (see text for details). A) Presentation of 7 symbols within the 4×6 checkerboard during memory encoding. B) The 7 symbols are randomly located at the bottom of the screen during memory retrieval.

The VMT is designed to measure episodic learning and delayed memory. The VMT consists of 7 symbols placed on a 4×6 checkerboard. After a practice session consisting of 2 symbols on a 2×2 checkerboard, the patient is presented with 7 symbols dispersed across a 4×6 checkerboard for 10 s (see Fig. 1A). The symbols are then removed from the checkerboard, placed at the bottom of the screen (see Fig. 1B) and the patient is instructed to move the symbols back onto the checkerboard using a stylus held in the dominant hand. Once the patient has finished placing all 7 symbols, the identical board pattern is redisplayed for 10 s. The patient is given a total of 5 trials. On each trial, a maximum score of 14 can be obtained. One point is provided if the patient correctly places any symbol in one of the 7 squares. Two points are awarded if both the location and symbol are placed in the correct square. A maximum score for the five learning trials is 70. If a perfect score is obtained on two consecutive trials (e.g., trials 3 and 4), the test is discontinued and a perfect score is allotted to the remaining trials (e.g., trial 5). With each subsequent administration, a unique display is presented to minimize practice effects. One of 6 different location patterns are randomly selected. Each of the 7 symbols are randomly drawn from one of 7 different semantic categories (fruits/vegetables, human activities, animals, plants, transportation vehicles, clothing, and tools) to minimize semantic clustering; each category has 6 exemplars for a total of 42 possible symbols. Thus, the chance of the same checkerboard pattern being presented consecutively isinfinitesimal.

### Processing speed test

**Fig. 2:**
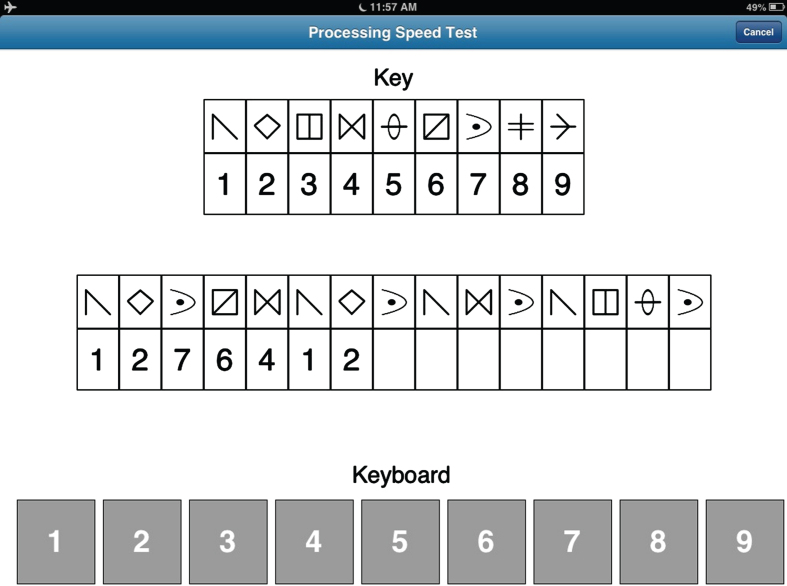
Screenshot of the Processing Speed Test (see text for details).

This module was designed to resemble the Symbol Digit Modalities Test [15]. The PST display (see Fig. 2) consists of a symbol-digit key at the top of the screen, which is randomly generated with each administration from a pool of 30 symbols. The middle row contains 15 symbols with empty boxes below each symbol. The participant is instructed to insert the appropriate digit below each symbol consistent with the key, moving automatically from left to right. Insertion is made by making a finger keypress applied to a keyboard, composed of the digits 1–9, located at the bottom of the screen. Once the participant completes the row, a new row of 15 symbols with empty boxes automatically appears. This process is repeated for 120 s, at which time the test is terminated. Both total correct and total incorrect responses are recorded. A practice trial, using a different key, is provided prior to the test trial.

The commercial version of the C3B is called *Cognition Chronicle^*TM*^* (Qr8 Health, Inc; Cleveland, OH).

## STUDY 1: NORMATIVE DATABASE

### Rationale

Cognitive test scores frequently correlate with demographic variables, such as age, education, sex, race, and ethnicity [18, 19]. Thus, the clinical meaningfulness of a particular raw score may be different depending on whether the raw score is obtained from a younger versus older patient, higher versus lower educated patient, as examples. One way to improve clinical interpretation is to administer the C3B to a sample of healthy individuals stratified across these demographic variables. Creation of such a normative database can yield regression-based statistics that enable an adjustment of the raw score to minimize the effects of demographic variables in test interpretation [20, 21]. The purpose of study 1, therefore, is to provide standardized regression equations for the two C3B test modules.

### Methods

To achieve geographic balance, 428 cognitively intact, healthy adults (ages 18 to 89 years) were recruited from four United States testing sites: Cleveland Clinic – Cleveland, OH; Cleveland Clinic – Las Vegas, NV; Kessler Institute for Rehabilitation - West Orange, NJ; and the University of California - San Diego, CA. Table 1 summarizes the demographic characteristics (age, sex, education) of the stratified sample. Stratification was verified by the absence of statistically significant associations between the three demographic variables. The sample included 73 African-Americans (AA; 17.1%), 28 Asians (6.5%), and 53 Hispanics (12.4%) (see Supplementary Table 1). Because of the relatively low number of Asians and Hispanics, these groups were not included as variables in the regression analyses.

**Table 1:** Demographic characteristics of normative sample

		Years of Education			
Sex	Age	9–12	13–15	16+	Total
Female	18–39	22	28	24	74
	40–59	17	25	24	66
	60–89	27	33	29	89
	*Total*	*66*	*86*	*77*	*229*
Male	18–39	22	24	23	69
	40–59	17	21	26	64
	60–89	11	21	34	66
	*Total*	*50*	*66*	*83*	*199*
Total	18–39	44	52	47	143
	40–59	34	46	50	130
	60–89	38	54	63	155
	*Total*	*116*	*152*	*160*	*428*

All testing was completed in a single session lasting less than 1 h. In addition to the two C3B tests, participants completed the MMSE [9] and the Beck Depression Inventory (BDI-II) [22]. Participants were excluded if the MMSE score was <28 and BDI was >9. Demographic and medical history information (including current medications) were also obtained to exclude potential participants meeting one or more of the exclusion criteria: 1) neurological illnesses/conditions; 2) medical illnesses/conditions that may affect brain function; 3) major psychiatric disturbance meeting DSM-IV Axis I criteria; 4) active substance abuse; and 5) current use of prescribed psychoactive medications (SSRIs and SNRIs were allowed). Participants received compensation for their time and travel expenses. The protocol was approved by the Institutional Review Boards at each of the four sites.

Exploratory analyses using Generalized Additive Models (GAM) were conducted to determine the relationship between each of the PST and VMT measures and age, sex, education, and race (AA versus non-AA). The relationships between neuroperformance and the continuous measures of age and education were described using natural splines. Fitting multivariate GAMs with increasing degrees of freedom for age and education enabled an examination of flexible non-linear functional relationships, which were tested for significance using ANOVA. The most parsimonious GAMs with statistically significant terms were identified as best reflecting the observed relationships. These models were subsequently translated into parametric linear regression equations to yield regression parameters for calculating the predicted neuroperformance scores required for adjusted z-scores. For the VMT, quadratic splines were used to capture the functional forms identified by the exploratory GAMs, with the best fitting models selected based on the model adjusted R-squared.

### Results

**Fig. 3:**
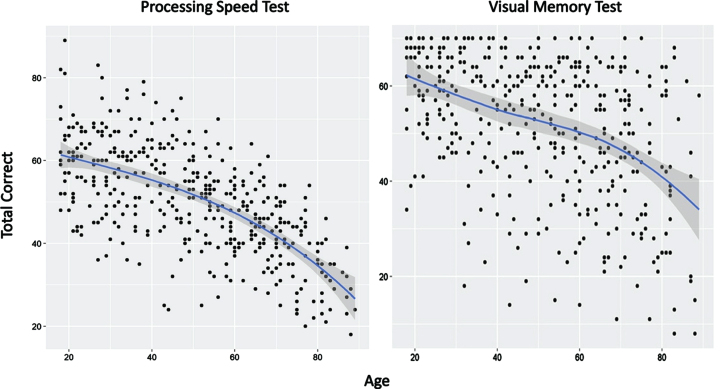
Effects of age on performance of the Processing Speed Test and Visual Memory Test. Blue line reflects best fit of the data (see text for details).

For the PST, there was a substantial 50% shared variance with demographic variables (R^2^ = 0.4963; Table 2). A simple quadratic polynomial showed an increasingly rapid decline in performance with increasing age (Fig. 3). The association between years of education and PST score was linear and positive, reflecting improved PST performance with increasing education. Male participants performed significantly worse than females and AAs performed significantly worse than non-AAs. Using the following formula, calculation of adjusted z-scores for the PST can be achieved by comparing the actual raw score to a calculated predicted score based on demographic variables (shown in brackets):

**Table 2:** Generalized additive models relating C3B tests and demographic variables

C3B Test	Intercept	Age	Non-Linear Age	Education	Sex	Race
*Processing Speed Test: Adjusted R^2^* = *0.4963; RMSE*						
= *8.958; Non-linear Age* = *Age^2^, centered on 50.26714*						
Estimate	65.2217	–0.4591	–0.0053	0.7999	–2.2132	–4.0893
SE	3.0794	0.0231	0.0013	0.1874	0.8782	1.2034
*t*	21.1800	–19.8570	–4.1670	4.2680	–2.5200	3.3980
*p*	**<0.0001**	**<0.0001**	**<0.0001**	**<0.0001**	**0.0121**	**<0.0001**
*Visual Memory Test: Adjusted R^2^* = *0.224; RMSE*						
= *12.879; Non-linear Age* = *(Age-71)^2^|Age*> *71 [(max(0,(age-71)))^2^]*						
Estimate	58.8785	–0.3118	–0.0329	0.7152	–	–5.6555
SE	4.2324	0.0372	0.0155	0.2685	–	1.7071
*t*	13.9110	–8.3790	–2.1260	2.6640	–	–3.3130
*p*	**<0.0001**	**<0.0001**	**0.0341**	**0.0080**	–	**0.0010**

Sex, 0 = Female, 1 = Male; Race, 0 = Non-African American, 1 = African-American; Education in Years (range = 0 to 20).

$$\begin{array}{cc} & \mathrm{Adjusted}\;Z-\mathrm{Score}=\\ & \;\;(\mathrm{Raw}\;\mathrm{Score}-[65.222+-0.4591*\mathrm{Age}\\ & \;\;+-0.0053*(\mathrm{Age}-50.2671{)}^{2}\\ & \;\;+0.7999*\mathrm{Education}+-2.213*\mathrm{Sex}+\\ & \;\;-4.089*\mathrm{Race}])/8.958 \end{array}$$

For the VMT, the amount of shared variance with demographic variables was significant but accounted for less than the amount of shared variance compared to the PST (R^2^ = 0.224; Table 2). A quadratic spline with a single knot at age 71 provided the best model fit (Fig. 3). This model showed a negative linear slope for participants under age 71 with no non-linear contributions to variance. For individuals aged 71 and older, VMT could be modeled with both linear and non-linear contributions. The association between years of education and VMT score was linear and positive, reflecting improved VMT scores with increasing education. Male and female participants performed similarly. AAs performed significantly worse than non-AAs. The adjusted z-sore formula for the VMT for individuals <71 years of age (calculation of predicted score shown in brackets):

$$\begin{array}{cc} & \mathrm{Adjusted}\;Z-\mathrm{Score}=\\ & \;\;(\mathrm{Raw}\;\mathrm{Score}-[58.879+-0.3118*\mathrm{Age}+\\ & \;\;0.715*\mathrm{Education}+-5.655*\mathrm{Race}])/12.879 \end{array}$$

For individuals ages 71 and older, the formula is:

$$\begin{array}{cc} & \mathrm{Adjusted}\;Z-\mathrm{Score}=\\ & \;\;\;(\mathrm{Raw}\;\mathrm{Score}-[58.879+-0.3118*\mathrm{Age}+\\ & \;\;\;-0.0329*(\mathrm{Age}-71{)}^{2}+0.715*\mathrm{Education}\\ & \;\;\;+-5.655*\mathrm{Race}])/12.879 \end{array}$$

### Discussion

The importance of using demographic data to adjust raw scores can be illustrated by the following example. Two hypothetical patients achieve identical raw scores on the PST and VMT (see Table 3). Patient 1 is a 30-year-old, Caucasian female with 16 years of education. Patient 2 is a 60-year-old, African-American male with 10 years of education. The adjusted z-score represents the difference between the patient’s raw score based on actual test performance and the raw score predicted solely by demographic data. This difference is divided by the root mean square error (RMSE) to create a z-score with a mean of 0 and a standard deviation of 1. Patient 1’s raw scores are >2 standard deviations below the predicted scores on both the PST and VMT, suggesting cognitive impairment. In contrast, Patient 2 is performing well within the normal range on both tests (i.e.,<1.0 standard deviation difference between the actual and predicted raw scores).

**Table 3:** Illustrative patient interpretations using adjusted Z-scores (see results for explanation)

Patient No.	Raw Score	Predicted Score	Adjusted Z-Score	Interpretation
*Processing Speed Test*				
1	36	59.8	–2.66	Impaired
2	36	31.4	0.51	WNL
*Visual Memory Test*				
1	33	61.0	–2.17	Impaired
2	33	42.1	–0.71	WNL

## STUDY 2: TEST-RETEST RELIABILITY AND PRACTICE EFFECTS

### Rationale

Important requirements for a cognitive screening tool include having minimal practice/learning effects and acceptable test-retest reliability. This study was designed to examine two-week practice effects and test-retest reliability of the two C3B test modules, VMT and PST.

### Methods

Thirty cognitively intact, healthy elders [males = 17 (56.7%); mean age = 73.5 years (SD = 5.3); mean education = 15.7 years (SD = 3.1); Blacks = 5 (16.7%) were recruited through advertisement flyers and community outreach events. Exclusion criteria were identical to those listed for Study 1 (Normative Study). Participants received compensation for their time and travel expenses; the protocol was approved by the Cleveland Clinic Institutional Review Board.

The VMT and PST were administered in a fixed order (VMT before PST) on two testing sessions separated by a two-week interval (mean days = 14.6 (SD = 2.0). Paired samples *t*-tests and intraclass correlation coefficients (ICC) were used to compare the PST (total number correct in 2 min) and VMT (total sum correct on trials 1–5) between the first and second administrations of the PST.

### Results

**Fig. 4:**
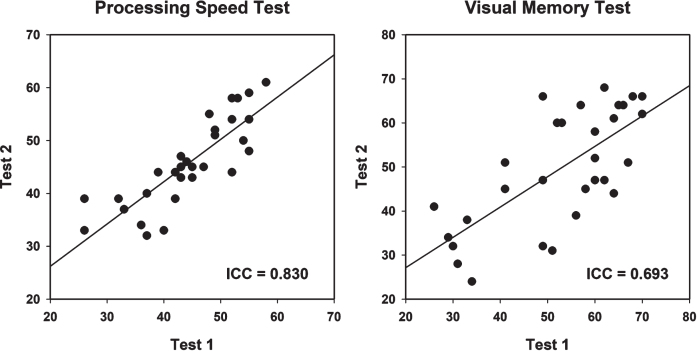
Test-retest reliability scatterplot with best fitting linear line for the Processing Speed Test and Visual Memory Test (see text for details). ICC, Intraclass Correlation.

Table 4 shows results of analyses comparing the first and second administrations of the C3B tests. There were no significant differences for either the VMT total correct of trials 1–5 or PST total correct (*p* = 0.12 and 0.14, respectively), indicating no appreciable learning effects across test sessions. Figure 4 shows scatterplots for the VMT and PST for the first and second test administrations. The ICCs for the PST was 0.830 and for the VMT was 0.693.

**Table 4:** Mean (SD) differences in PST and VMT performance over a 14-day interval

	Test 1		Test 2		Difference			
	Mean	SD	Mean	SD	T1 - T2	*t*	*p*	Cohen’s d
PST	44.4	8.6	45.7	8.2	–1.3	–1.52	0.14	–0.278
VMT	52.6	13.6	49.6	13.3	3	1.59	0.12	–0.291

### Discussion

These results indicate that the two C3B test modules do not exhibit practice effects over a 2-week interval. In addition, both modules exhibit acceptably high levels of test-retest reliability.

## STUDY 3: SENSITIVITY TO MILD COGNITIVE IMPAIRMENT

### Rationale

This study examined the sensitivity of the C3B in distinguishing between patients with MCI and demographically matched healthy controls. The sensitivity of the C3B test modules were also compared to standardized neuropsychological (NP) instruments used in dementia clinical practice. Finally, this study compared the sensitivity and specificity of the C3B to the Mini-Cog [13], one of the most commonly used cognitive screening instrument in primary care clinical settings.

### Methods

MCI patients (*n* = 30) were recruited from the Cleveland Alzheimer’s Disease Research Center and met clinical, neuropsychological, neuroimaging, and biomarker criteria for MCI based on a multi-disciplinary ADRC diagnostic consensus conference. The 30 healthy controls (HC) that participated in Study 2 served as the comparison sample for this study; results of their first testing session were used for this study. Participants received compensation for their time and travel expenses; the protocol was approved by the Cleveland Clinic Institutional Review Board.

Participants self-administered the C3B and completed the following technician-administered standardized NP tests: Hopkins Verbal Learning Test-Revised (HVLT-R [23]), Brief Visual Memory Test-Revised (BVMT-R [16]), and WAIS-IV Coding subtest (Coding [24]). In addition, both groups were administered the Mini-Cog.

Independent *t*-tests were conducted to compare the MCI and HC groups on the C3B and NP measures, with Cohen’s D being used to determine effect size. In addition, signal detection analyses were used to generate the area-under-the-curve (AUC) of the receiver operating characteristic (ROC) curve.

The comparison of the sensitivity and specificity of the C3B and Mini-Cog required the application of cutoff scores for the two measures. The Mini-Cog has a range of scores from 0 to 5; impairment is clinically defined by a score <3. For the C3B, the appropriate cutoff scores were determined from the signal detection analyses applied to the demographically-adjusted z-scores derived from the normative study (Study 1). Impairment was defined by the lower score of the VMT and PST rather than an average of the two scores, since some MCI patients may experience greater deficits in episodic memory than processing speed, whereas the opposite pattern may be observed for other MCI patients. Youden’s J statistic [25] was calculated to compare the performance of the C3B and Mini-Cog as dichotomous diagnostic tests; an acceptable Youden index is 50% or higher.

### Results

No differences were observed between the two groups on sex [MCI = 18 males (60%); HC = 17 males (56.7%); *χ*^2^ = 0.01, *p* = 0.79] and race [MCI = 6 African-Americans (20%); HC = 5 African-Americans (16.7%); *χ*^2^ = 1.02, *p* = 0.60]. The MCI and HC groups were comparable in age and education (Table 5); as expected, the two groups were significantly different on the MoCA(Table 5).

**Table 5:** Demographic and cognitive test results for the MCI and HC groups

	MCI		HC				
	Mean	SD	Mean	SD	*t*	*p*	Cohen’s d
Age	75.07	7.23	73.53	5.25	–0.94	0.351	–0.243
Education	15.77	2.90	15.70	3.06	–0.09	0.931	–0.022
MoCA	20.03	2.97	28.33	1.56	13.50	<0.001	3.516
C3B Tests							
PST	28.61	11.27	44.27	8.48	6.01	<0.001	1.579
VMT	26.57	14.97	51.63	14.18	6.66	<0.001	1.719
Traditional NP Tests							
BVMT-R	7.67	4.82	22.33	6.91	9.53	<0.001	2.461
HVLT-R	14.83	4.51	26.83	5.13	9.53	<0.001	2.481
Coding	9.76	3.76	16.77	13.24	2.74	0.008	0.715

**Fig. 5:**
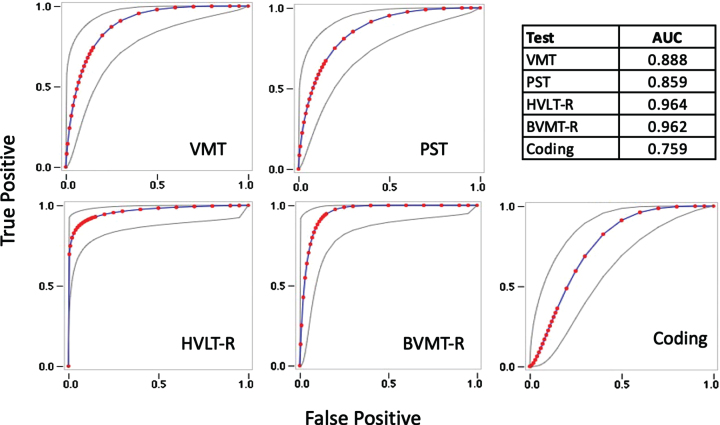
Receiver Operating Characteristic curves with Areas Under the Curve (AUC) estimates for the Visual Memory Test (VMT), Processing Speed Test (PST), Hopkins Verbal Learning Test-Revised (HVLT-R), Brief Visual Memory Test-Revised (BVMT-R), and WAIS-IV Coding subtest (Coding).

The MCI group performed significantly worse than the HC group on the C3B and NP tests (Table 5). Cohen’s d indicated strong and comparable effect sizes for the episodic memory measures (VMT, BVMT-R, and HVLT-R), whereas on measures of processing speed, the PST demonstrated a greater effect size compared to Coding. The AUC of the ROC curves were high for the C3B measures and comparable to the technician-administered NP tests (Fig. 5).

Based on the signal detection analyses, the optimal cutoff scores for the C3B measures were *z*< –0.4. With a clinically accepted cutoff score <3, the Mini-Cog identified only 45% (*n* = 13) of the MCI patients as impaired, whereas the C3B correctly identified 90% (*n* = 27) as impaired (Table 6). The overall Youden Index was 0.45 for the Mini-Cog, indicating an inability of this screening test to discriminate MCI patients from cognitively normal individuals. In contrast, the Youden Index was 0.73 on the C3B, indicating excellent ability to discriminate the two groups.

**Table 6:** Comparison of sensitivity and specificity of the C3B and MiniCog

Cutoff score	TP	FP	TN	FN	Sensitivity	Specificity	Youden Index
MiniCog <3	13 (45%)	0 (0%)	30 (100%)	16 (55%)	0.45	1.00	0.45
PST or VMT z < –0.4	27 (90%)	5 (17%)	25 (83%)	3 (10%)	0.90	0.83	0.73

TP, true positive; FP, false positive; TN, true negative; FN, false negative.

### Discussion

This study demonstrates that the 10-min C3B screening tests exhibit high levels of sensitivity in discriminating MCI patients from healthy individuals. For episodic memory, the VMT is comparable in sensitivity to the BVMT-R and HVLT-R, whereas, for processing speed, the PST demonstrates slightly better sensitivity than Coding. In a direct comparison between cognitive screening measures, C3B and Mini-Cog, the C3B demonstrated a high level of discriminability with minimal false positives and false negatives. Surprisingly, the Mini-Cog, a commonly used screening test used in primary care settings, did not meet minimal statistical criteria for discriminating MCI from controls using the clinically established cutoff score <3. The Mini-Cog identified only 45% of MCI patients as impaired.

## STUDY 4: EFFECTS OF WAITING ROOM VERSUS QUIET ROOM ON C3B PERFORMANCE

### Rationale

Neuropsychological examinations are typically conducted in a private room to maximize cognitive test performance by avoiding the potentially negative impact of attentional distractions, such as speech or environmental noises. Most primary care clinics do not have the luxury of having a dedicated quiet room to conduct cognitive screening. This study was designed to compare C3B test performance completed in a busy hospital waiting room with performance completed in a quiet, private room.

### Methods

Participants were 29 cognitively intact older adults [16 males (55.2%); mean age = 72.0 (SD = 5.5); mean education = 16.7 years (SD = 1.9)], who were recruited using methods described in Study 1. Participants were administered the C3B twice, 1 h apart, in each location, in counterbalanced order. Active noise-cancelling headphones were used to minimize extraneous, potentially distracting noises. Paired samples *t*-tests were used to compare the total score of the PST and VMT between administrations in the private room and waiting room. Participants received compensation for their time and travel expenses; the protocol was approved by the Cleveland Clinic Institutional Review Board.

### Results

**Fig. 6:**
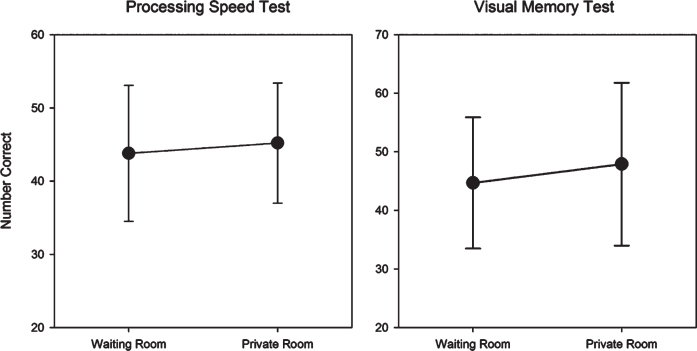
Mean (SD) waiting room and private room raw scores for the Processing Speed Test and Visual Memory Test.

No significant differences were observed in performance by testing location for the PST (*t* = 1.15, *p* = 0.26) and VMT (*t* = 1.77, *p* = 0.09) (see Fig. 6).

### Discussion

Results suggest that when participants are provided with active noise-cancelling headphones, sound distractions are minimized and C3B test performance can be validly obtained in a waiting room environment.

## STUDY 5: INTEGRATION OF THE C3B INTO A PRIMARY CARE CLINICAL SETTING

### Rationale

The self-administered C3B was designed to enable routine cognitive screening examinations of older patients (age >65) in a primary care setting with minimal disruption of clinical workflow. This study provides data on a C3B demonstration project. There were three primary goals: 1) examine completion rates for the self-administered and unsupervised C3B subtests; 2) assess patient satisfaction in taking the C3B as part of their routine clinical care; and 3) examine the relationship between participants’ memory ratings and the two objective C3B module scores.

### Methods

The C3B was administered in the waiting room to 470 consecutive primary care patients prior to their Medicare Annual Wellness Visit at the Cleveland Clinic Twinsburg Health and Surgery Center in Twinsburg, Ohio (Blazenka Skugor, Medical Director). The data collected from the iPads was transferred to the cloud where raw scores were converted to z-scores to adjust for demographics using the regression-based norms described in Study 1. Both raw and z-scores were inserted into the electronic medical record (EMR) such that the professional caregiver would have the results during the same patient clinic visit. After completion of the C3B, patients completed a 6-item survey to determine their level of satisfaction with taking the C3B. Immediately prior to the C3B administration, patients provided a subjective assessment of their memory functioning using the Cognitive Change Index (CCI) [26], a scale used in the Alzheimer’s Disease Neuroimaging Initiative project. Both the patient satisfaction survey and CCI were self-administered on the same iPad used for C3B self-administration. This study was considered part of routine clinical care; as such, patients were not required to sign a written informed consent to self-administer the C3B.

### Results

The 470 patients had a mean age of 73.8 (range 65–99) and a mean education of 14.6 (range 2–20); the sample was 71% female and 83% white. Incompletion rates for the self-administered and unsupervised C3B subtests were low: 4.9% for the PST and 7.4% for the VMT. Sixteen patients (3.4%) did not complete the C3B due to frustration and 3 patients (0.6%) refused; the remainder did not finish due to device issues or discontinuation by the provider.

**Fig. 7:**
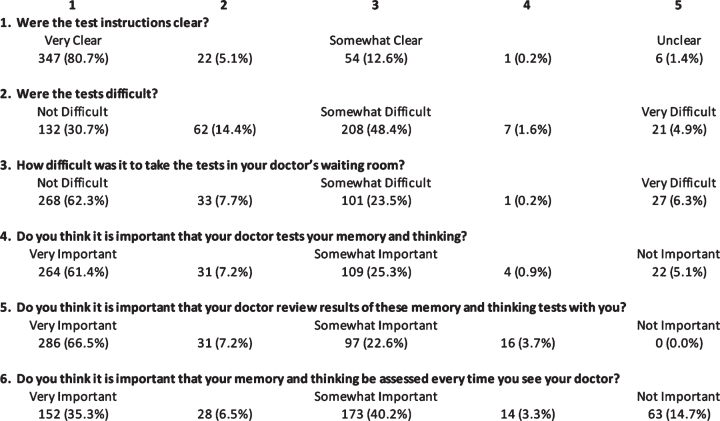
Results of a patient survey consisting of six 5-point Likert scale questions.

Figure 7 reports results of the 5-point patient satisfaction survey administered to 430 patients: 98.4% of patients reported the test instructions to be very clear to somewhat clear (ratings 1–3 collapsed); 93.5% thought the C3B tests were not difficult to somewhat difficult (1–3); 94.0% indicated that it was very important to somewhat important (1–3) that their provider test their memory and thinking; 96.3% thought that it was very important to somewhat important (1–3) that the provider reviewed results of the cognitive testing with the patient; and 82% expressed the opinion that annual assessments were very important to somewhat important (1–3).

Total score on the CCI correlated non-significantly with the PST (*r* = 0.021) and VMT (*r* = 0.101).

### Discussion

Three conclusions can be drawn from results of this study: 1) the C3B has a very high rate of completion (>92%) despite the fact that the PST and VMT are self-administered and patients are not supervised; 2) the patient satisfaction survey indicates that the C3B tests are well-tolerated and designed and patients think the cognitive test results are important for their clinical care; and 3) the relationship between subjective ratings of memory impairment correlate poorly with objective measures of episodic memory and processing speed, underscoring the need to conduct dementia screenings using objective cognitive tests.

## OVERALL DISCUSSION

Results from this series of studies have shown that: 1) the C3B raw scores can be converted to demographically-adjusted, z-scores based on regression-based equations derived from a large stratified sample of healthy adults, thus providing a more accurate clinical interpretation of the C3B screening (Study 1); 2) the C3B test modules have acceptably high levels of test-retest reliability and minimal practice effects, enabling an accurate assessment of longitudinal changes in episodic memory and processing speed (Study 2); 3) the C3B is able to discriminate MCI patients from demographically-matched healthy individuals at a level that is comparable to standardized cognitive tests used in neuropsychological clinical practice and is superior to the Mini-Cog, a screening test frequently used in primary care clinical practice (Study 3); 4) using sound-attenuated headphones, the C3B can provide accurate data even when self-administered in a distracting testing environment, such as a clinic waiting room (Study 4); and 5), in a demonstration project conducted within a primary care setting, the C3B modules had a high completion rate (>92%) in older adults who may have minimal computer skills; the older adults rated the C3B modules as well-tolerated and important for their clinical care; and subjective ratings of memory loss correlated poorly with objective measures of episodic memory and processing speed, underscoring the need to conduct dementia screenings using objective cognitive tests (Study 5). Overall, this series of studies support the use of the C3B for cognitive screening in a primary care setting.

Whereas there exist reliable and valid computerized cognitive test batteries, the C3B has six key innovations that allow it to stand out. *First*, the C3B modules were designed to be self-administered by mimicking the role of a technician. All instructions are auditory to avoid cluttering the screen and confusing the test taker. Both the VMT and PST use practice tests that include visual and auditory feedback; individuals are not allowed to take the test without having a thorough understanding of the test instructions during the practice sessions. These design features have resulted in the very high self-administration completion rates in an unsupervised setting in older patients, some of whom have minimal computer skills. In a previous study [27], we compared the self-administration completion rates of the C3B to the CogState Brief Battery (CSBB) [28, 29]; without technician supervision, the incompletion rate was 1.3% for the C3B and 13.8% for the CSBB. The latter rate is unacceptably high for a clinical environment.

*Second*, the C3B was specifically designed to be fully integrated into a primary care practice setting. In our demonstration project (Study 5), primary care patients completed the C3B in the waiting room immediately prior to being seen by their professional caregiver. Raw C3B data were instantaneously transferred to the cloud, where adjusted scores, based on a normative database (Study 1), were computed and automatically transferred directly into the EMR before the patient was seen by their physician. Thus, the primary care professional caregiver can subsequently discuss the results of the C3B screening with the patient during the same visit and make appropriate referrals for follow-up examinations in low-scoring patients.

*Third*, the C3B test modules were designed to detect patients at the early MCI stage rather than detect patients already meeting criteria for dementia. The modules were designed to be more difficult than most dementia screening examinations, which exhibit ceiling effects and are insensitive to the more subtle deficits exhibited by MCI patients. Not surprisingly, the C3B was superior to Mini-Cog in discriminating MCI patients from healthy older adults. Using a standard clinic cutoff score of <3, the MiniCog detected only 45% of MCI patients as impaired, whereas the C3B detected 90% as impaired. Thus, the C3B demonstrated superior sensitivity to the Mini-Cog without substantial differences in specificity, which was comparably high for both screening tests. Not surprisingly, the C3B modules exhibited comparable sensitivity and specificity to standardized neuropsychological tests used in dementia clinical practice, since the C3B modules were designed to have as wide a range of difficulty as standardized neuropsychological tests to enable detection of patients at the early MCI stage.

*Fourth*, the C3B was designed to be brief (10 min). More importantly, self-administration improves workflow since primary care clinics are no longer required to allocate staff to administer and score cognitive screening tests since this is accomplished automatically by the C3B software. As an example, in a typical Mini-Cog administration, it takes 5 min to administer the test, 2 min to score, and 1 min to enter the score into the EMR. Thus, the Mini-Cog requires approximately 8 min of valuable clinic personnel time per patient evaluation. In contrast, the C3B requires less than a minute to provide the patient with the iPad and headphones.

*Fifth*, the C3B modules use visual stimuli that consist of single digit numbers (1–9) and symbols. This avoids the problems associated with translating verbal stimuli into different languages. On verbal list learning tests, the frequency of usage of a word can influence its memorability. The only verbal stimuli are the auditory test instructions, which can be readily translated into various languages. The C3B is currently translated into Spanish and 5 other languages.

*Sixth*, the C3B was designed to scale to any size primary care practice. While primarily designed to be EMR integrated, a standalone version of the C3B has been developed. This version requires the primary care provider to manually enter raw and z scores into the medical record.

The VMT was initially developed with a delayed memory trial, but this was dropped in the final version due to low test-retest reliability, as is common on scores derived from a single trial. Delayed memory is critical on most cognitive screening tests (MMSE, 3MS, MoCA, Mini-Cog) because the number of items to be recalled is typically three. On immediate recall, patients can readily recall three items because this is well within the capacity of working memory, thus rendering immediate recall ineffective in documenting problems with episodic memory. In contrast, the VMT uses seven stimuli and the patient must recall both the item and the location. Because patients must recall 14 bits of information on each of five trials, the task demands are well beyond working memory capacity, enabling the VMT to engage episodic memory mechanisms. In addition, because the final score involves the summation of five trials rather than one, the measure has acceptable test-retest reliability (S2).

In S5, only 16 of 470 consecutive primary care patients (3.4%) were unable to complete the C3B in the waiting room due to “frustration.” It is unclear whether patients were unable to complete the C3B due to cognitive impairment, computer-related test anxiety, disruptions in clinic workflow, testing in a distracting waiting room environment, or other factors. In any case, the overall number of patients who were unable to self-administer the C3B is remarkably low.

These studies must be viewed in light of several limitations. *First*, as is true of all cognitive screening instruments, the C3B does not replace more comprehensive neuropsychological evaluations, which are required for addressing complex clinical issues related to differential diagnosis, treatment decisions, and case management. *Second*, the regression equations were derived from a US sample of healthy individuals. Such equations may not apply to other countries/cultures even when the test instructions are translated into a native language. Additional normative studies may be required to provide more accurate diagnostic data for a particular country/culture. *Third*, the normative sample of 428 individuals could be larger by focusing on increasing the numbers of Hispanics and raising the percent of individuals with lower educational attainment (0–12 years) from 27.1% (S1) to the nationwide average of 37.6% based on the 2020 census. *Fourth*, the sensitivity/specificity data were derived from 30 MCI patients and 30 demographically comparable healthy adults. The patients were already diagnosed with MCI after referral to a dementia clinic. A larger scale study is needed to determine the accuracy of detecting MCI in patients who underwent C3B screening in a primary care setting. Using signal detection methods, more accurate cutoff scores could be derived that simultaneously minimize both false positives and false negatives. Such a study is currently underway. *Fifth*, S5 examined C3B completion rates and patient satisfaction with C3B screening in a suburban primary care clinic. Additional work is needed to determine completion rates and patient satisfaction in less affluent urban and rural primary care settings. *Finally*, longitudinal research is needed to determine whether the C3B is sensitive to cognitive decline over time.

To summarize, our findings indicate that the C3B meets criteria established by the 2017 NIA workshop [14] that a computerized cognitive screening tool be validated, self-administered, and fully integrated into the primary care clinical workflow for detecting MCI, early AD, and other related dementias. The C3B would appear to be an optimal digital screening tool to meet practical recommendations for a timely and accurate diagnosis of MCI and early AD in a primary care setting [6] to enable modification of known risk factors that account for nearly half of all dementias [7].

## Supplementary Material

Supplementary Material

## Data Availability

The data supporting the findings of this study are available on request from the corresponding author. The data are not publicly available due to privacy or ethical restrictions.
