# Supplementary material for: Cleveland Clinic Cognitive Battery (C3B): Normative, Reliability, and Validation Studies of a Self-Administered Computerized Tool for Screening Cognitive Dysfunction in Primary Care
Source: J Alzheimers Dis. 2023 Apr 4;92(3):1051–66. doi: 10.3233/JAD-220929 (PMC10116145; doi:10.3233/JAD-220929)
Supplement: Supplementary Material [file jad-92-jad220929-s001.pdf]

Supplementary Material

Cleveland Clinic Cognitive Battery (C3B): Normative, Reliability, and Validation Studies of a Self-Administered Computerized Tool for Screening Cognitive Dysfunction in Primary Care

Supplementary Table 1. Race and Ethnicity Enrollment by Testing Site.

		RACE						
**Location**		**American Indian/ Alaska Native**	**Asian**	**Black/ African-American**	**More than one race**	**Other**	**White**	**Total**
CC-Cleveland	Count	2.000	13.000	35.000	1.000	0.000	115.000	166.000
	%	1.205%	7.831%	21.084%	0.602%	0.000%	69.277%	100.000%
CC-Las Vegas	Count	2.000	6.000	14.000	2.000	0.000	103.000	127.000
	%	1.575%	4.724%	11.024%	1.575%	0.000%	81.102%	100.000%
Kessler	Count	1.000	6.000	21.000	1.000	4.000	56.000	89.000
	%	1.124%	6.742%	23.596%	1.124%	4.494%	62.921%	100.000%
UCSD	Count	0.000	3.000	3.000	1.000	4.000	35.000	46.000
	%	0.000%	6.522%	6.522%	2.174%	8.696%	76.087%	100.000%
Total	Count	5.000	28.000	73.000	5.000	8.000	309.000	428.000
	%	1.168%	6.542%	17.056%	1.168%	1.869%	72.196%	100.000%

		ETHNICITY		
**Location**		**Hispanic or Latino**	**NOT Hispanic or Latino**	**Total**
CC-Cleveland	Count	14.000	152.000	166.000
	%	8.434%	91.566%	100.000%
CC-Las Vegas	Count	20.000	107.000	127.000
	%	15.748%	84.252%	100.000%
Kessler	Count	8.000	81.000	89.000
	%	8.989%	91.011%	100.000%
UCSD	Count	11.000	35.000	46.000
	%	23.913%	76.087%	100.000%
Total	Count	53.000	375.000	428.000
	%	12.383%	87.617%	100.000%
